# Experiences of a digital health intervention for young people exposed to technology assisted sexual abuse: a qualitative study

**DOI:** 10.1186/s12888-024-05605-6

**Published:** 2024-03-28

**Authors:** Ethel Quayle, Amanda Larkin, Matthias Schwannauer, Filippo Varese, Kim Cartwright, Prathiba Chitsabesan, Victoria Green, Gillian Radford, Cathy Richards, Sara Shafi, Pauline Whelan, Cindy Chan, William Hewins, Alice Newton, Erica Niebauer, Marina Sandys, Jennifer Ward, Sandra Bucci

**Affiliations:** 1 Department of Clinical and Health Psychology, School of Health in Social Science, University of Edinburgh, Edinburgh, UK; 2 Greater Manchester Mental Health NHS Foundation Trust, Manchester, UK; 3 NHS Lothian, Edinburgh, UK; 4 Division of Psychology and Mental Health, School of Health Sciences, Faculty of Biology, Medicine and Health, Manchester Academic Health Science, The University of Manchester, Manchester, UK; 5 Pennine Care NHS Foundation Trust, Ashton-under-Lyne, UK; 6 Marie Collins Foundation, Manchester, UK

**Keywords:** Online child sexual abuse, Child and adolescent psychiatric care, Digital health intervention, Qualitative, Coproduction

## Abstract

**Background:**

There is growing evidence that Technology Assisted Sexual Abuse (TASA) represents a serious problem for large numbers of children. To date, there are very few evidence-based interventions available to young people (YP) after they have been exposed to this form of abuse, and access to support services remains a challenge. Digital tools such as smartphones have the potential to increase access to mental health support and may provide an opportunity for YP to both manage their distress and reduce the possibility of further victimization. The current study explores the acceptability of a digital health intervention (DHI; the i-Minds app) which is a theory-driven, co-produced, mentalization-based DHI designed for YP aged 12–18 who have experienced TASA.

**Methods:**

Semi-structured interviews were conducted with 15 YP recruited through Child and Adolescent Mental Health Services, a Sexual Assault Referral Centre and an e-therapy provider who had access to the i-Minds app as part of a feasibility clinical trial. Interviews focused on the acceptability and usability of i-Minds and were coded to themes based on the Acceptability of Healthcare Interventions framework.

**Results:**

All participants found the i-Minds app acceptable. Many aspects of the app were seen as enjoyable and useful in helping YP understand their abuse, manage feelings, and change behavior. The app was seen as usable and easy to navigate, but for some participants the level of text was problematic and aspects of the content was, at times, emotionally distressing at times.

**Conclusions:**

The i-Minds app is useful in the management of TASA and helping change some risk-related vulnerabilities. The app was designed, developed and evaluated with YP who had experienced TASA and this may account for the high levels of acceptability seen.

**Trial registration:**

The trial was registered on the ISRCTN registry on the 12/04/2022 as i-Minds: a digital intervention for young people exposed to online sexual abuse (ISRCTN43130832).

## Background

Young people’s lives are increasingly interwoven with technology use [1, 2] such that we need to think of ‘cybersystems’ as part of the developmental ecology of childhood [3–5]. While online sexual risks do not always result in harm for young people (YP), there is evidence that in general population samples of young adults, lifetime prevalence of Technology Assisted Abuse (TASA) is a significant problem [6, 7]. While prevalence rates vary across different forms of TASA (image-based sexual abuse, self-produced child sexual abuse images, non-consensual sexting; online grooming by adults, revenge pornography, sextortion, and commercial sexual exploitation), in a recent US nationally representative online survey of young adults aged 18–28, the overall prevalence of TASA was 15.6%. YP aged 13–17 were more likely to be targets across all groups and a larger percentage of perpetrators were other YP [7]. However, prevalence rates appear to be higher in YP from high-risk populations [8]. For example, in this study, 20% of adolescents referred to a US Child Advocacy Center reported online sexual solicitation with individuals met online. There is growing evidence of the negative emotional impact of TASA, including self-harm [9, 10] depression and anxiety, self-blame and shame [11]. The misuse of sexual images has a particularly negative valence for YP [12]. While the Internet does not make YP more vulnerable to TASA, it may leave already vulnerable YP more accessible to victimization [13].

Multiple factors are likely to be involved in vulnerability to being exposed to TASA. Vulnerable YP, or those living with offline risks or disadvantage, are more exposed to online risks and in turn find themselves more likely to experience harm and less able to find support [14]. A publication from the US online survey [7] found that YP who were cisgender female, non-heterosexual with parents who had experienced only high-school education were more likely to experience TASA, but early offline sexual abuse remained the strongest predictor of TASA [15].

One potential risk factor for TASA is the ability of YP to accurately estimate others’ intentions and motivations when online [16, 17]. This ability to understand one’s own and other’s minds and mental states is known as mentalization [18]. Mentalizing promotes the development of a stable sense of self and helps young people consolidate their understanding of motivations and feelings related to the self / others and subsequently improves social and interpersonal functioning. While mentalizing is related to resilience in response to stressors, the capacity to mentalize can be undermined by stress and arousal [19]. Older children may show more adaptive mentalization than younger children and similarly typically developing children are better at mentalizing than those who are seen as atypical [20]. High levels of online activity may also be related to a reduced ability to mentalize and be associated with high levels of emotional distress [21]; during the COVID-19 pandemic, epistemic trust (the ability to evaluate incoming information from the social world as accurate, reliable, and relevant [22]) and mentalizing were negatively associated with perceived stress and emotion dysregulation [18].

People’s assumptions about the intentions and motives of others are usually based on the verbal and nonverbal cues from real-life interactions. When communicating online, signs of empathy and understanding are not communicated as clearly and can be more obfuscated [23]. Difficulty mentalizing can influence people’s ability to correctly evaluate risk and trust when interacting online [16, 24] and might therefore represent a valuable target for a mentalization-informed intervention aimed at reducing risk in YP who have already been exposed to TASA. Difficulties mentalizing have been linked to greater vulnerability to a range of mental health problems that are common among TASA survivors [25]. Indeed, a recent systematic review [26] highlighted mentalization-based therapy (MBT) as a promising treatment approach across a range of clinical presentations [27]. The distinctive nature of TASA is recognized in current National Institute for Clinical Excellence (NICE) guidelines for responding to child abuse and neglect [28]. Indeed, there are no evidence-based interventions for improving the mental health and well-being of YP who have experienced TASA; NICE has recommended further research on the efficacy of interventions aimed at improving well-being and relationships and preventing further harm following internet-facilitated abuse; the efficacy of such interventions remains an unmet research need [29, 30].

### The role of digital interventions

Digital Health Interventions (DHI) represent feasible, acceptable, and potentially beneficial options for YP with physical and mental health needs on waiting lists [31]. Long waiting times in CAMHS indicate that there are significant delays in help being offered, preventing timely access to support, potential exacerbation of problems brought about by TASA exposure, and increased risk for repeated victimization in the interim. Recent systematic reviews and meta-analyses have demonstrated that DHIs represent effective treatment options for improving the mental health and well-being of YP across a range of problems [32, 33] along with promoting healthy romantic relationships in adolescents [34] and improving sexual health [35]. However, it has also been noted that while digital interventions can be effective substitutes or supplements to traditional mental health interventions with adolescents, only a small number of existing DHIs are evidence based [36]. Existing feasibility, acceptability, and efficacy studies of DHIs indicate that they are acceptable across genders [37], can impact behavior as well as mood [38], and are safe for vulnerable YP [39]. In addition, adolescents with sexual and gender minority identities do report positive attitudes towards brief DHIs which may address some of the existing barriers to traditional treatment interventions for these YP [40].

Engagement of YP in the design, development and evaluation of DHIs has been seen as critical [34, 41, 42] and should prioritize the voices of YP [43]. A systematic review [44] identified the different modes of delivery used in DHIs for YP, explored the factors that influence usage and implementation, and investigated how interventions have been evaluated and whether YP engage with DHIs. Engagement is commonly referred to as the active involvement of participants with the intervention, also described in previous literature as participation, adherence, noncompliance, or resistance [41]. This knowledge is crucial to support the development and evaluation of DHIs that are acceptable and feasible in CAMHS. The review identified themes which encompassed factors such as suitability, usability, and acceptability of the DHIs and motivation, capability, and opportunity for the YP using DHIs. YP prefer DHIs with features such as videos, limited text, ability to personalize, ability to connect with others, and options to receive text message reminders. The findings of this review suggested a high average retention rate of 79% across studies involving a variety of DHIs.

Sekhon et al. provided an overview of the acceptability of DHIs and from this developed a theoretical framework [45]. Their definition of acceptability is “… a multifaceted construct that reflects the extent to which people delivering or receiving a healthcare intervention consider it to be appropriate, based on anticipated or experienced cognitive and emotional response to the intervention.” p 9. They identified seven component constructs: affective attitude, burden, perceived effectiveness, ethicality, intervention coherence, opportunity costs and self-efficacy. This framework was used to inform a qualitative analysis of stakeholder perspectives on the acceptability of a DHI (My Journey 3) for supported self-management in early intervention services for psychosis [46]. In addition, they included a separate theme reflecting suggestions on how to improve content, design, or delivery of the app.

The current study is part of a larger program of work to codevelop and test the feasibility, acceptability and safety of the i-Minds app, a theory-driven, coproduced, mentalization-based DHI designed for YP aged 12–18 who had experienced TASA [30]. The purpose of the research was to understand how participants experienced the i-Minds app and its acceptability. We also addressed the perceived benefits or problems in using i-Minds and the impact of i-Minds on the YP’s life, the barriers and facilitators to using the i-Minds app and suggested improvements to the app. To our knowledge, this is the first study to examine qualitatively YP’s views about a mentalization-based DHI targeting TASA.

## Methods

### Setting

This qualitative study was nested within the i-Minds non-randomized feasibility clinical trial of a digital intervention to improve mental health and interpersonal resilience in YP who have experienced TASA [29]. Participants were approached directly by the research team to participate in a qualitative exit interview after a 6-week intervention exposure window. Both participants who did and did not complete the feasibility clinical trial in its entirety were asked to participate in the interview. Participants were selected according to a sampling framework to capture varied demographics, experiences of TASA, and levels of engagement with the i-Minds app. Of the 45 YP recruited into i- Minds feasibility clinical trial in two UK NHS sites (Manchester, Edinburgh), 15 took part in the post intervention interviews.

### Participants

Participants who had participated in the feasibility clinical trial of i-Minds (Bucci et al., *in submission*) and who consented to participate in qualitative exit interviews after taking part in the trial were eligible to be interviewed. Participants were invited to this quality study at the point when information was provided, and consent obtained for the larger i-Minds feasibility clinical trial. Participants aged 12–15 years recruited from the English NHS recruitment site were given a bespoke participant information sheet to give to at least one of their parents/caregivers who had the ability to opt their child out of taking part in the study. Participants aged 12–15 years recruited from the Scottish site were asked if they would like their parents/carers to be given a participant information sheet. Inclusion criteria were: (i) aged 12 to 18 years; (ii) exposed to TASA reporting associated distress and receiving support from National Health Service CAMHS, Sexual Assault Referral Centre or an e-therapy provider; (iii) willing to use an YP-TASA app; (iv) proficient in speaking and writing English; and (v) capacity to consent; (vi) consent to providing their username to the research team (e-therapy provider participants only).

### Intervention

The i-Minds app is a 6-week modular intervention underpinned by mentalization principles and designed to be used as a standalone platform without restrictions. We followed the overall structure and content of a mentalization-based manual developed previously by members of the research team [27] and adapted across different digital media to include scenarios related to TASA that the YP could interact with. The aim of the intervention was to help YP understand the motives of both adults and peers, protect them from future abuse and leave them feeling more confident in interpersonal interactions that may be ambiguous or challenging. The content of the app was organized into four key areas: (i) mentalization; (ii) psychoeducation about TASA; (iii) emotional and mental health; and (iv) trauma. Cross-cutting links were provided within topics along with a repository of available resources that could be addressed from the home screen. The app was available on the participant’s smartphone or a loaned device. Participants were required to complete the mentalization module before progressing onto other areas of the app. App development was supported through participatory consultations to enable users to influence the design and functionality of the app. No limits were set on how often, or where, the app should be used or with whom it could be discussed with; however, daily prompts invited YP to check in with the app if they had not done so that day. Participants were reimbursed for data usage.

### Procedures

All YP people who consented to use the app were invited for an interview following the 6-week intervention period. Participants could choose whether to be interviewed in-person or remotely and were reminded that this was optional and would explore their experience of taking part in the i-Minds trial and its acceptability. A small financial incentive was given upon interview completion. A semi-structured interview schedule was developed by the research team and an expert by experience. Questions and prompts were designed to assess feasibility and acceptability of taking part in the i-Minds trial, using the i-Minds app, and possible barriers and facilitators to its use. Questions included, ‘Please could you describe what it was like using the i-Minds app?’; ‘Try to remember the last time you used i- Minds. What did you do?’; ‘You were given i-Minds to use over a 6-week period. Did anything change in how you used the app over this time?’; ‘How could we have made the app better for you?’. Interview topics covered: (i) usability and acceptability of i-Minds; (ii) benefits or problems in using i-Minds?; (iii) impact of i-Minds on the YP’s life; (iv) barriers and facilitators to using the i-Minds app; and (v) improvements to the app.

### Analysis

Encrypted audio-recordings were made of the interviews and then downloaded and transcribed. Analysis was supported by the end-to-end encrypted software application Dedoose for qualitative and mixed methods research [47]. A predominantly inductive approach was adopted. Data was open-coded, and meanings based on the interpretations made by respondents was emphasized. Deductive analysis was also employed to ensure that the open coding allowed for the identification of themes that were meaningful to the research questions posed. This thematic framework included the seven constructs relating to the acceptability of the intervention: affective attitude, burden, ethicality, intervention coherence, opportunity costs, perceived effectiveness, and self-efficacy (Table 1). Therefore, both semantic and latent codes were used and we followed the proposed recursive and iterative six-stage analytical process to facilitate coding and theme-identification: (i) familiarization with the data; (ii) generating initial codes; (iii) generating themes; (iv) reviewing potential themes; (v) defining and naming themes, and (vi) producing the report [48]. An additional theme that related to how i- Minds might be improved was added.

**Table 1:** Thematic framework adapted from the acceptability of healthcare interventions theoretical framework

Construct	Description
Affective attitude	How an individual feels about using the i- Minds app
Burden	The perceived amount of effort that is required to participate in i-Minds
Ethicality	The extent to which the i-Minds app has good fit with an individual’s value system
Intervention coherence	The extent to which the participant used the i-Minds app and how it works
Opportunity costs	The extent to which benefits, profits or values must be given up to engage in i-Minds
Perceived effectiveness	The extent to which the i-Minds app is perceived as likely to achieve its purpose
Self-efficacy	The participant’s confidence that they can perform the behavior(s) required to participate in i-Minds

## Results

Participant demographics can be seen in Table 2. Participants were mainly white females aged 15.3 years without previous experience of using a mental health app. Interviews lasted between 29 and 59 min (m = 44).

**Table 2:** Participant demographics

	Participants *N* (%)
**Gender**	
Female	11 (73.3)
Male	01 (6.7)
Non-binary/not stated	03 (20.0)
**Age, years– mean (range)**	15.3 (12 to 18)
**Ethnicity**	
White British	15 (100)
Highest completed level of education*	
Primary school	09 (60%)
Secondary school (up to GCSEs)	06 (40%)
**Time receiving support from services in months– mean (range)**	10.3 (1 to 50)
Index of multiple deprivation decile** - mean (range)	05.4 (1 to 10)
**Previously used a mental health app**	
Yes	05 (33.3)
No	10 (66.7)

*All but 2 were still in education but had not completed their final state exams; **1 most deprived– 10 least deprived

### Theme 1: affective attitude

The response to taking part in the i-Minds trial and using the app was largely positive. YP overall found the app helpful for the difficulties they had experienced:

*“But I think now that I went through the process of using it for a while I feel quite positive feelings towards it, like a thankfulness. Because I think it helped” (ED-007).*

*“I think it was one of those apps and it would be really good for people who have maybe just had a lot of that type of stuff [happen]. Because it talks a lot about how they might be feeling and all that, they felt understood. I thought that was quite nice” (ED-002).*

For many participants, the app left them feeling that they were not on their own, and what had happened to them was more common than they thought:

*“I don’t know, it made me realise I’m not alone, I’m not the only person that this happened to, and that there is help out there, yes” (EDI-017).*

*“Like, it made me think that I’m not alone in what happened to me and that my situation is also similar to other people’s and that I shouldn’t like dwell on the fact that I might be alone in it, when I know people have had the same experience” (MAN-002).*

Participants described feeling less isolated and more positive about themselves:

*“Yeah, I think it’s made me think a little bit more positively about myself as I know that I’m not the only one who’s experienced these kind of things” (KOO-001).*

They also talked about the design of the app being rewarding and engaging:

*“I just found it, like, really easy to use, and it was quite entertaining” (EDI-013).*

The design of the i- Minds app included feedback about how the various exercises were used and this was represented by a tree, with each topic representing branches of a tree, and sub-topics as smaller branches. As the user works through the material a leaf is added to the tree such that the tree appears to grow. There was a lot of feedback in the interviews about how rewarding this was:

*“I feel, like, the tree was, like, really good because you get a leaf every time you do something. So, you’ve achieved something every time you do it” (EDI-005).*

*“… and the trees with the leaves…like it was sort of like a reward at the end” (EDI-006).*

There were also suggestions about how to build on this as a design feature:

*“Maybe with the tree it can grow, or something, I don’t know. Like, it can grow from, like, a caterpillar to a butterfly, or it can go from, like, an egg to a big bird– something like that. And that’s nice” (EDI-013).*

However, for some participants using the app was triggering and associated with some level of distress; this was particularly the case in relation to the content of some sections:

*“I really can’t put my…like my finger on it, but there was something where I was like, actually, this is enough. That’s enough for today” (EDI-008).*

*“…the aim of it [the video material] was to make you feel like it’s not just happening to you, but in reality, it sort of brought it all back” (ED-006).*

Participants referred to the tensions about appropriateness of the content in relation to their feelings of distress, but over time this tension reduced the more they interacted with the app content:

*“I think this is a good thing and a bad thing, but all of the tree stuff was quite difficult. I felt it brought up things that I don’t really like thinking about, that was stressful, but I think ultimately, it’s good to do that” (ED-007).*

*“I definitely really struggled at first with it, and then I like found different ways to just manage it and that and saw that helped with that” (MAN-003).*

### Theme 2: burden

Only a few participants talked about how effortful they found the app to use with certain aspects of the app viewed as unwelcome. This particularly related to the amount of text participants were required to read:

*“Probably because…I’m not completely sure, but the way it’s set up makes it feel like it’s just going to be a lot of paragraphs of…like, I didn’t use it, but it just felt like it was going to end up being a lot of paragraphs about stuff, and I didn’t have…at the time, I didn’t like reading” (EDI-013).*

*“I didn’t really like the amount of reading there that there was” (KOO-003).*

Being able to choose when to use the app meant that there was variation in the length of time spent using the i-Minds app both between participants as well as for individuals. The ability to control this (despite prompts from the app) seemed if anything to reduce the perceived burden:

*“I think at first I thought I have to use this quite a lot because I’m part of this study type thing, but then if I’m using it when I don’t need it, it’s not going to be as important and use it when I do need it, so I calmed down with using it too much and decided to start using it when I needed it” (MAN-008).*

No constraints were placed on how often, when or where the app should be used and there were considerable differences across the sample with some YP scheduling regular times each day to open the app and others using it as needed, or when they remembered. The app was also used in private spaces (bedrooms) and times (at night when on their own), whereas some YP shared the content with friends or family or used it on the bus after school.

### Theme 3: ethicality

There was little in our data that talked to the alignment between the i-Minds app and individual value systems. All participants interviewed had used their own smartphones during the trial which they used regularly for socializing and seeking information. Some, but not all, had used other DHIs and felt that digital tools were a good way of accessing help. No participants mentioned feeling that this was a poor substitute for in-person engagement with their therapist. Importantly, none of the users felt that the app for was unsafe for them to use:

*“It felt like how the data that was being collected was being used was made really clear. And what was and wasn’t being shared was made really clear. So, it felt like a very safe app to use” (EDI-007).*

It was also clear that while some of the content about TASA was quite explicit and at certain times caused some feelings of distress in some participants (as in Theme 1), none of the YP felt that this was inappropriate or should be modified:

*“Yes, it was quite a tough topic, but it talks about it in a good way” (ED-002).*

*“Traumatic, no. It was more just like I feel like everyone’s been babying it down a wee bit… and it was kind of like actually this is…this is right. This is true” (EDI-008).*

### Theme 4: intervention coherence

All users had a clear idea of what i-Minds was and how to use it. What was appreciated was the support from the research team when participants initially started to use the app:

*“I think it’s well explained like in an overview what the app was about and how to use it really well. And so I knew what I was meant to do. I found it very clear, and I think the app itself also it has the instructions, and the introduction bit, I found that very clear and very helpful” (EDI-07).*

*“It was just like, when you were showing me where everything was, and how to use it and what there was on it, about the quotes and stuff” (MAN-007).*

There were frequent references to the fact that it was straightforward to use and appeared to participants to be coherent in what it had to offer while still giving users autonomy over how they could navigate the content:

*“It was a good experience. It wasn’t, like, difficult to navigate and there were different things for what different people are comfortable with doing” (MAN-008).*

It also seemed that for most participants the content of the app included material that they could relate to, and which appeared relevant to them:

*“I think I probably learned more about my own experiences, and it was really informative” (MAN-003).*

### Theme 5: opportunity costs

Participants’ views on whether anything had to be given up to engage with the app suggested that this was not really an issue for them and there was easy movement between using their smartphone for social activities and using the i-Minds app. There were even instances where YP talked about sharing the app with their friends at times when they were together. Several participants noted that there were occasions when they moved away from scrolling through their social media to using i-Minds and suggested that it was a more interesting thing to do. Participants were asked about issues that related to how safe they felt the app to be, and universally it was felt that privacy or potential misuse of data was not a problem:

*“Yeah, I had a really good understanding of what I was getting into, and I liked that at any point I could like opt out and change my mind, that was helpful” (EDI-006).*

Some participants specifically mentioned that there were benefits to being part of the study that were a bonus rather than a cost and this specifically related to financial reward:

*“Well, I think the getting paid, it was like, I wouldn’t have cared if I didn’t or not, but I think it was sort of like a reward sort of thing” (EDI-006).*

*“Honestly, it’s because D says it’s £20 pound when you do the interview and at that time, I was fresh at my new job and I didn’t have any money in my bank account, so being completely honest, I just thought it was just a way to make money” (MAN-008).*

### Theme 6: the perceived effectiveness of the app

Participants consistently reported that they found the i-Minds app helpful in the ways that it allowed them to see things from the perspective of other people, to manage their own thoughts and feelings, and to make changes in both their online and offline behavior. These changes were specifically linked with different sections of the app as well as by virtue of simply having access to the app:

“I think it’s made me think more about how it changed me for the better, and how I’m a lot more safer now, rather than thinking about the negative” (MAN-007).

For some users, seeing things from another’s perspective had an impact not only on understanding one’s own thoughts but it impacted on how users felt about themselves:

*“Yeah, I think it changed how I think about things, and it kind of makes me want to be more polite to people, and I think that’s a big thing, because I think, it sort of shows you that everyone’s going through stuff, and I’m not the only one going through stuff and I should be nice to people. But I also feel like when you’re nice to other people, it makes you feel better” (MAN-007).*

*“I used, like the exercises they’d done. Any time I am in a situation, I do take it from, like, someone else’s perspective for how I deal with the situation I’m in. So taking it from, like, someone else’s perspective and then taking it back into my self-experience is very useful” (EDI-013).*

Recognizing and managing feelings was one of the main objectives of the app and this was reflected in the interviews:

*“I’d say it’s really hard to put into words. But I think I learned a bit more about - this is hard to describe– I think it’s because I don’t like thinking about any of the bad things that have happened to me, especially in regard to the online things. So I just don’t think about them and then when I don’t really have words or more than a very sort of basic understanding of as something happens. And I think that sort of using the app, for me, made me feel like I could explain those things better and explain my feelings about them and why I did it and how I’m feeling about it now” (EDI-007).*

However, some of the most telling reflections of the impact of the app related to changes in technology-related behavior:

*“I think it was just, like before when I was talking to people and that things like I knew I was doing but I didn’t really care– it’s difficult to explain, but it just felt like I was doing something, but I was doing it without thinking. I felt like I was doing it, but it wasn’t me. It was like a bit out of body, I felt like I wasn’t really there when I was doing it and I think after using that app I was able to properly be more into touch with myself when the need for attention came out and say this is not the way to get it “ (EDI-007).*

*“Yes, well, I feel like when I’d find myself in some of these situations, I’d want validation from just people in general and so it’s stopped me doing that, really, because I now realise actually this is how these kinds of things have happened to me, so maybe don’t do that again” (ED-017).*

### Theme 7: self-efficacy

Confidence levels for all participants remained high throughout the intervention. In the main, participants said the app was easy to use and that they did not require additional support from either the research team or their clinician:

*“It was easy to use. It’s not the most complex thing in the world, you know? It’s kind of point and click to where you want to go, I guess” (EDI-017).*

*“The courses. Well, the sections, really. It was like, you know, the journey part of it. It just felt like, because it was set up a bit like a journey, going through, like, it did help a lot with taking things slowly. Because usually before that, I did try to heal from a lot of stuff, but I’ve gone through it at a fast pace, because I wanted to get there fast, but the app really helped just put things in sections and go through it slowly…” (EDI-013).*

Self-efficacy was also shown in how participants made choices about the use of different functions in relation to the app. For example, there was a daily prompt which many found helpful, but some turned it off, and while others had technical problems with prompts, they felt confident in being able to sort these out on their own or were quick to ask for technical assistance.

For a small number of participants, some content in the app was not easy to find and they referred to how the functionality might be impacted upon by emotional arousal:

*“But I found it bit difficult to find some of the things. And there were a few buttons to press, so I think if you were like going there because you wanted to calm down after… so that, I think that might be a bit, not bad, but just a bit harder to navigate, I suppose” (EDI-007).*

YP made only passing reference to using the app with their therapist, seeing it as something they used more in a standalone manner rather than integrating it as part of any routine therapeutic support they were receiving:

*“No, not really. When we spoke [the therapist] would just ask, how is it going? I would say, yes, it is going okay” (EDI-002).*

*“Not really, to be honest. I like mentioned it, but never really went in depth about it” (EDI-016).*

*“It’s none of their business, really” (KOO-003).*

Participants were aware that their therapist had referred them to the trial, and therefore knew about the app, but no specific guidance had been given about whether they should or should not discuss with them how they were using it.

### Theme 8: suggestions for improvement

Participants discussed how the app might be improved. Suggestions included adding more content that related to specific forms of TASA, making the app more interactive, and the ability to customize the app more to make it more rewarding to engage with:

*“So, it would be a cute character. It would be like an animal or a little guy, and then its role would be to go through the journey with you, and to encourage you to come back to the app and to do your next bit of the course. And it would be encouraging, and it would be nice” (EDI-013).*

Interestingly, YP talked about missing the app when it was no longer available and for some participants there was regret or sadness expressed about no longer having access to the app. This led to suggestions that there should have been the option to use the i- Minds app for longer than the period specified:

*“Just a bit sad. Not necessarily sad, just sad and happy about what I learned from it, but sad that it wasn’t there anymore I would say” (EDI-007).*

There were also changing patterns in how the app was reported being used over the 6-week period, with some participants saying that the i-Minds app needs to be available to YP when they are actually exposed to TASA rather than sometime later.

## Discussion

The aim of this study was to understand how participants experienced the i-Minds app and its acceptability. Fifteen YP took part following a 6-week intervention period. Most participants found the app to be acceptable and easy to use. They were able to navigate their way through the content without the need for additional support from the research team or their supporting clinician. Of interest was that all four key content areas of the app were mentioned in terms of everyday use, and each content area served different functions, informing YP about TASA and helping them identify commonalities in their experiences, being able to understand their own feelings and those of others, and distress tolerance. Participants said that using the app reduced feelings of isolation and created a positive sense of achievement and reward, especially when the appearance of another leaf on the tree graphic included in the app appeared. However, for a small number of YP, the content of the app was at times distressing and triggering; although, this distress was short lived, and the content was seen as a necessary part of processing the experience of TASA. While it appeared that some YP talked to family members about this, there was less explicit discussion of bringing their feelings of distress into their therapy sessions. This is not to say that it did not happen, but it was not discussed in the interviews. YP consistently talked about the effectiveness of the app; in particular, explicit references were made by some YP to how it changed both online and offline behaviors. Clearly this was not the case for all; some YP said that they had already started to change their online behaviors prior to using the app. However, this remains an important finding as behavior change has been difficult to evidence in most traditional safety education programs (as opposed to changing awareness or attitudes; [49]. One study that did indicate change following a brief DHI educational intervention with school children demonstrated a reduction in sexual interaction behaviors (such as sharing or sending sexual photos or videos of themselves) with adults engaged in online grooming [50], which was not the case for the control condition. These results demonstrated that a brief digital intervention may be effective in not only increasing knowledge about one form of TASA but also in reducing engagement with online perpetrators.

All participants interviewed used their own smartphone to access the app. This reflects current smartphone usage patterns in the UK [2] for YP within this age group who used handheld devices as well as tablet and gaming consoles to socialize, download and create content. Of interest was how fluid their use of different applications was such that they might be chatting on WhatsApp and then moving onto the i-Minds app. In this sense, the use of the app did not seem to be burdensome in that participants were all active technology users, felt confident in being able to manage technical problems when experienced, and felt that the app was straightforward to use and made sense. What was a burden for some was the amount of text in the app, which developers should bear in mind when developing future DHIs for YP. YP managed this by simply skipping the text and accessing video content instead. YP also said the app could be improved by making it more interactive and adding more abuse-specific content. There were individual preferences in relation to more or less videos and personal stories, but the involvement of YP and young adults with and without lived experience at each stage of app development appeared to ensure that the content and format was seen as relevant and acceptable [44].

Outside of completing the mandatory mentalization domain in the i-Minds app, no other restrictions on how the content could be accessed/used was in place. Similar to other findings [44], participants liked the availability of videos, the personalization features, the diary function, prompts and the self-soothing resources. They reported enjoying the interactive aspect of the app and would have liked more of this and less text, as well as expressing a preference for being able to communicate with other YP with some experiences. They also referred to the app being rewarding to use and appreciated the small financial benefits that came from participating in the study. Other studies have noted that YP may be more likely to complete a brief DHI in the context of paid research than in an unpaid context [51]; this may prove to be problematic in relation to non-supported use. However, the flexibility of being able to use i-Minds in a way that met individual needs and abilities may also reduce demographic disparities by minimizing the demands made on the user [52].

The i- Minds app was reportedly safe to use, and no concerns were expressed about privacy or security of information by YP, which is different to was has been found in the wider literature [53]. However, for some YP, there was a suggestion that while the content of the app was seen as both relevant and effective, it would have been more helpful if it had been available to them closer to the time when TASA had been experienced. This remains something of a challenge as for many of the YP within the study the experience and consequences of TASA were not necessarily the reason for accessing CAMHS or other support services and disclosure of what had happened may not have been made until sometime after the event. There is consistent evidence that practitioners do not routinely ask questions about TASA and that there are no evidenced-based practices to support them [29, 54–56]. This might suggest that i-Minds may be more relevant when made available through other youth-focused services or in schools. This may also serve to empower YP and enable help-seeking, increase ease of access, allow for anonymity and reduce the potential stress of face-to-face encounters [53].

Reflective of other DHIs, it appears that users developed a sense of connection and alliance with the i-Minds app, and described missing the app when access to it stopped. The concept of a Digital Therapeutic Alliance (DTA; [57] has received attention in recent years given the finding that users report relational feelings that reflect the concept of therapeutic alliance when using DHIs [58, 59].

### Strengths and limitations

To our knowledge, this is the first acceptability study of a DHI developed to support YP following exposure to TASA. We have identified potential barriers for implementation, such as the timing of access to the i-Minds app in relation to TASA, and what features increased the likelihood of it being used. Central to the design and implementation of the app was the involvement of people who had experienced TASA; this is a strength of the study and increased the likelihood that participants felt that they had the skills to use the app and that i-Minds was perceived as likely to achieve its purpose [43].

Findings need to be considered alongside some limitations. All the YP recruited were part of the larger i-Minds feasibility study; as the participants in this study all agreed to be interviewed, this may not reflect all the views of the larger group of YP who took part in the i-Minds trial. All participants had access to the app through their own smartphone. While this was seen in a positive light by YP and allowed for the app to be embedded in the flow of the user’s daily (digital) life, it may also be the case that these YP were more technologically capable and confident than other users and have a positive view of technology [60]. While we sought diversity in the sample, most participants were female, and all identified as ‘White British’. There is clearly a need to address this in further research. As access to the app was limited to a 6-week intervention period, we also have no way of knowing whether there would have been changes in how the app was used over a longer period. Previous experience with DHIs, negative experiences with traditional mental health services or when disclosing experiences of TASA, and socially desirable responses during interviews, might have influenced participants’ expressed views.

## Conclusions

This qualitative study shows that the i-Minds app is acceptable to YP who have experienced TASA and is safe to use. That said, participants suggested ways to improve the app, mainly via having less text to read and more video and interactive content. Whilst, for some, the app content was triggering at times, this was short-lived and viewed as a necessary part of processing their experience of TASA. We call for more applied intervention research, using both controlled trial and qualitative methods, to understand the impact of, and offer support with, the experience of TASA in YP, either through digital or non-digital formats. The efficacy of interventions that could improve well-being and prevent further harm in YP exposed to TASA remains an unmet research need.

## Data Availability

Due to the sensitive nature of the topic of this study and consent procedures used for data sharing, open access to the data is not possible. Therefore, requests to access the datasets should be directed to sandra.bucci@manchester.ac.uk.

## Abbreviations

YP: Young People
TASA: Technology Assisted Child Abuse
MBT: mentalization-based therapy
CAMHS: Child and Adolescent Mental Health Services
NICE: National Institute for Clinical Excellence
DHI: Digital Health Intervention
HIV: human immunodeficiency virus
NHS: National Health Service
DTA: Digital Therapeutic Alliance
